# Adverse effects of atrazine on blood parameters, biochemical profile and genotoxicity of snow trout (*Schizothorax plagiostomus*)

**DOI:** 10.1016/j.sjbs.2021.01.001

**Published:** 2021-01-07

**Authors:** Naveed Akhtar, Muhammad Fiaz Khan, Sadia Tabassum, Eman Zahran

**Affiliations:** a Department of Zoology, Hazara University Mansehra, Pakistan; b Department of Internal Medicine, Infectious and Fish Diseases, Faculty of Veterinary Medicine, Mansoura University, Egypt

**Keywords:** Snow trout, Hematology, Biochemistry, Genotoxicity, Comet Assay

## Abstract

This study was conducted to evaluate the adverse effects of atrazine on hematology, biochemistry and genotoxicity of snow trout (*Schizothorax plagiostomus*). Almost all treated groups presented considerably (*P* < 0.05) lesser values of hematocrit, hemoglobin, WBC, RBC, MCH, MCHC, monocytes and lymphocytes while significantly higher values of HCT and platelets are observed. A Significant decrease is observed in sodium, calcium, potassium, phosphorous, triglycerides, creatinine, urea, and total protein contents whereas, a significant increase is observed in cholesterol and glucose level. Significant (*P* < 0.05) alterations are observed in enzyme activities of all treated groups. DNA damage was observed at the concentrations (2–4 ppm). Results showed that Comet assay is reliable for evaluating the toxicity and is helpful in environmental monitoring programs.

## 1 Introduction

Atrazine is among the widely used herbicide in agricultural techniques for corn, sorghum and sugarcane. Atrazine was primarily introduced as a phytotoxic agent to stops photosynthesis through the opposition with plastoquinone II at its compulsory site progress the electron transport in photosystem II in target plants (Lebaron et al., 2008, Solomon et al., 2008).

Atrazine might be abstemiously lethal to aquatic fauna (Solomon et al., 2008). Various studies have shown its oxidative anxiety in many fish fauna (Blahová et al., 2013, Mela et al., 2013), variations in performance (Plhalova et al., 2012), or changes in biochemistry once exposed to severe, sublethal, or lethal concentrations. Harmful effects are reported about atrazine on productivity (Tillitt et al., 2010), resistant reaction (Kreutz et al., 2012), or decontaminating system (Fu et al., 2013). Many tissues like liver, kidney, gill, or other fish organs are affected after exposure to atrazine (Paulino et al., 2012). Severe dose of exposure can result in indications like changes in fish performance and pathological function (Solomon et al., 2008).

Atrazine is among the highly used chemical for controlling unwanted plants in corn or crops, like green vegetables (Cui et al., 2012). It has the chance to mix with water due to its well moment within soil (Waring and Moore, 2004). Hussein et al. (1996) reported that atrazine extents to water due to immediacies of agronomic nations of aquatic places, or sometimes due to miss handling in specific environments. When they reach such an environment, they are not breakable by microorganisms (Gamble et al., 1983). However, (WHO, 1996) reported their breakdown in shallow seawater through light and microbial activity and the life of 20–50 days at 20–25 °C under in vitro conditions whereas an increase is observed in its activity at low temperature (USEPA, 1988).

Due to current environmental problems, fish is been highly affected as it is directly exposed to ecological pollution which shows the natural possessions of ecological effluence in waters. An initial investigative study of hematology might be helpful in evaluating initial threatening symbols of insecticide effects (Pant et al., 1987). In Pakistan, atrazine is still a popular herbicide used against wide-ranging leaf wildflowers and greenswards. The impact of atrazine is high which has resulted in the stimulation of studies related to its toxicity in aquatic fauna. Therefore this research was conducted to evaluate the effects of atrazine on hematology, biochemistry and genotoxicity in *Schizothorax plagiostomus*.

## 2 Materials and methods

### 2.1 Concentration of herbicide

Five experimental glass aquariums were used in which one was used as a control and in the remaining four aquariums different doses (1 ppm, 2 ppm, 3 ppm and 4 ppm) of atrazine were used. After the introduction of atrazine, fish behavior and mortality was observed.

### 2.2 Collection and acclimatization of fish

Specimens of *Schizothorax plagiostomus* were collected from River Swat and were transferred in oxygen bags to the laboratory of Zoology Department, Hazara University Mansehra. Fishes were treated via KMnO4 to remove any sort of impurity. Fish specimens were kept in glass aquariums and were acclimatized for 14 days under laboratory conditions and water was replaced after every 24 h.

### 2.3 Blood collection and analysis

After 96 h of chemical exposure, five fishes were randomly collected from each aquarium and were anesthetized in clove oil to collect blood. Blood was collected by puncturing the caudal fin using a 1 mL disposable syringe.

### 2.4 Hematology

To study hematology, blood was collected from fish in EDTA tubes. Hemoglobin (Hb) amount was accomplished by following the cyano-methemoglobin technique having a spectrophotometer of 540 nm absorbance (spectrophotometric technique). To determine packed cell volume (PCV), the technique of microhematocrit was used. Using dacie‘s solution, red blood cells (RBC) and white blood cells (WBC) were determined (Blaxhall and Daisley, 2013). Documentation of erythrocyte catalogs containing mean corpuscular volume, mean corpuscular hemoglobin and mean corpuscular hemoglobin concentration (MCHC) were resolute by (Haney et al., 1992) process. Furthermore, variance total was executed for leukocyte proportion documentation. Blood smears were marked using 5% Giemsa solution and a description of every cell proportion was calculated in one hundred cells.

### 2.5 Biochemistry

For biochemical analysis, blood was collected in Gel ETDA tubes were used and collected blood was centrifuged to separate plasma. Plasma was isolated and biochemical parameters were determined using Chem Reader SBA-733 Plus (Semi-auto Chemistry analyzer, Advanced Japanese Technology). Biochemical parameters included in this study were; Electrolytes (Calcium, Potassium, Phosphorus and Sodium), Metabolites (Total Protein, Glucose, Creatinine, Urea, Cholesterol and Triglycerides) and Enzymes (Lactate Dehydrogenase (LDH) Alanine, Alkaline Phosphatase (ALP), Aspartate Aminotransferase (AST) and Aminotransferase (ALT)).

### 2.6 Genotoxicity

The genotoxicity was executed by following the technique used by (Singh et al., 1988). Blood samples were adulterated in saline solution of 1000 μl. Using 10 uL of saline solution slides were prepared and (0.5%) of low melting agarose of 120 μl at 37 °C. In lysis solution consisting (1 mL of Triton X-100, 10 mL of DMSO and 89 mL of lysing solution stock, pH 10.0 - stock solution: 2.5 M of NaCl, 100 mM of EDTA100, 10 mM to 1 L of Tris) slides were kept for 60 min in the refrigerator. Slides after 60 min, slides were kept in a horizontal electrophoresis system at 25 V, 300 mA for 20 min. For about 15 min slides were neutralized with 0.4 M of Tris having pH 7.5, and stable in ethanol for 10 min. Those cells in which no DNA damage occurred move consistently, whereas those cells in which DNA damage had occurred, they show fragments of dissimilar masses, and minor cells move quicker in electrophoresis making the tail of a comet.

### 2.7 Statistical analysis

Data was analyzed with the help of SPSS software (Version 24.0). The comparison was made among all of the four experimental groups using one-way ANOVA. Variables were stated as means and standard deviations. p < 0.05 was considered as statistically significant.

## 3 Results

### 3.1 Fish behavior

In the control group, no behavioral changes were observed, whereas experimental groups presented irregular behavior like reduced responses, irregular swimming and loss of equilibrium. Mortality was observed only in the experimental group IV having the concentration of atrazine (4 ppm).

### 3.2 Hematological profile of blood

After the introduction of fishes to herbicide noteworthy alterations were observed in hematological parameters of all experimental groups. Those exposed to higher concentrations were highly affected. A Significant decrease was observed in WBCs counts of all treated groups. A Non-Significant decline was observed in erythrocyte count in experimental group IV that consequently led to a major decline in hemoglobin amount while slight changes were observed in hematocrit level in experimental group IV compared to the control group. A significant decline was observed in lymphocytes and monocytes counts in experimental group I-IV, whereas a decrease was observed in neutrophils in all experimental groups. Detailed analysis of hematological parameters of *Schizothorax plagiostomus* is given in Table 1.

**Table 1:** Detail hematological parameters of *Schizothorax plagiostomus.*

**Hematological Parameters**	**0 ppm**	**1 ppm**	**2 ppm**	**3 ppm**	**4 ppm**
WBCs (10^3/^mm^3^)	2.89 ± 0.017	2.6 ± 0.04^** Δ^	2.52 ± 0.03** ^Δ^	2.46 ± 0.03** ^Δ^	2.42 ± 0.04** ^Δ^
RBCs (10^6/ml^)	3.8 ± 0.47	3.2 ± 0.31** ^Δ^	2.8 ± 0.30** ^Δ^	2.3 ± 0.28** ^Δ^	1.91 ± 0.31** ^Δ^
Hb (g/dL)	12.5 ± 0.50	11.8 ± 0.34** ^Δ^	10.4 ± 0.51** ^Δ^	8.45 ± 0.54** ^Δ^	7.44 ± 0.50** ^Δ^
HCT (%)	36.0 ± 0.94	44.2 ± 0.98** ^Δ^	41.0 ± 1.23** ^Δ^	30.6 ± 0.98** ^Δ^	28.2 ± 0.92** ^Δ^
MCV%	96.7 ± 10.5	112.5 ± 13.7**	127.8 ± 18.8**	132.5 ± 15.2**	145.6 ± 14.3
MCH %	34.0 ± 0.89	27.0 ± 0.74** ^Δ^	23.1 ± 1.38** ^Δ^	22.4 ± 1.27** ^Δ^	22.5 ± 1.35** ^Δ^
MCHC %	32.8 ± 2.8	46.4 ± 4.4**	42.0 ± 9.6	39.0 ± 3.87	37.7 ± 2.81
Platelets (10^3/mm3^)	3.68 ± 0.05	6.2 ± 0.08** ^Δ^	7.68 ± 0.12** ^Δ^	8.6 ± 0.05** ^Δ^	11.5 ± 0.02** ^Δ^
Monocytes%	4.62 ± 1.16	4.87 ± 1.2 ^Δ^	3.6 ± 0.51 ^Δ^	4.24 ± 1.5 ^Δ^	2.46 ± 0.58 ^Δ^
Lymphocytes%	66.7 ± 1.5	58.4 ± 1.4** ^Δ^	54.1 ± 1.7** ^Δ^	50.5 ± 1.9** ^Δ^	48.8 ± 1.34** ^Δ^
Neutrophils%	25.1 ± 1.74	38.7 ± 1.52^** Δ^	49.5 ± 0.88** ^Δ^	42.7 ± 2.3** ^Δ^	48.7 ± 1.15** ^Δ^

Unit of measurements: WBCs (10^3/^mm^3^), RBCs (10^6/ml^), Hb (g/dL), HCT (%), MCV (%), MCH (%) MCHC (%), Platelets (10^3/mm3^). Values are expressed as Mean ± SD (n = 5 fish per treatment). Mean with ** expresses significant difference (p < 0.05) while Δ shows significant intergroup difference.

### 3.3 Biochemical profile of blood plasma

After 96 h of chemical exposure, significant changes were observed in the level of calcium, phosphorus, total protein, glucose, LDH, ALP, AST and ALT. A significant decrease is observed in all electrolytes levels. A significant decrease is observed in triglycerides, urea and protein whereas cholesterol, creatinine and glucose activity increased in all experimental groups. A significant increase was observed in ALP, ALT, AST and LDH activity. Details of all biochemical parameters are given in Table 2.

**Table 2:** Summarized biochemical parameters of *Schizothorax plagiostomus.*

**Electrolytes**					
**Parameters**	0 ppm	1 ppm	2 ppm	3 ppm	4 ppm
Sodium	13.1 ± 0.26	12.4 ± 0.45	12.0 ± 0.32**	11.4 ± 0.14**	11.3 ± 0.21**
Potassium	12.4 ± 0.51	11.21 ± 0.32** ^Δ^	8.6 ± 0.54** ^Δ^	7.42 ± 0.53** ^Δ^	6.03 ± 0.67** ^Δ^
Calcium(mg/dl)	13.1 ± 0.27	11.8 ± 0.45	11.6 ± 0.31**	12.3 ± 0.15**	10.8 ± 0.24**
Phosphorous(mg/dl)	17.5 ± 0.54	14.2 ± 0.32** ^Δ^	10.4 ± 0.33** ^Δ^	8.8 ± 0.15** ^Δ^	5.8 ± 0.14** ^Δ^

**Metabolites**					
Triglycerides(mg/dl)	241.6 ± 3.5	228.2 ± 1.4** ^Δ^	184.2 ± 1.12** ^Δ^	162.1 ± 1.6** ^Δ^	141.8 ± 2.2** ^Δ^
Cholesterol(mg/dl)	231.5 ± 2.4	235.1 ± 3.8 ^Δ^	251.8 ± 1.16** ^Δ^	263.4 ± 7.6** ^Δ^	274.5 ± 2.3** ^Δ^
Urea(mg/dl)	13.2 ± 1.3	12.5 ± 2.4 ^Δ^	8.9 ± 1.4** ^Δ^	7.5 ± 1.3** ^Δ^	7.6 ± 1.2** ^Δ^
Creatinine	0.05 ± 0.01	0.035 ± 0.006 Δ	0.15 ± 0.01** Δ	0.32 ± 0.01** Δ	0.45 ± 0.04** Δ
Glucose(mg/dl)	133.4 ± 2.5	144.1 ± 3.5** ^Δ^	144.0 ± 1.6** ^Δ^	163.5 ± 1.3** ^Δ^	175.3 ± 1.7** ^Δ^
Total Protein(g/dl)	4.1 ± 0.53	3.6 ± 0.25 ^Δ^	2.6 ± 0.17** ^Δ^	2.6 ± 0.14** ^Δ^	2.8 ± 0.16** ^Δ^

**Enzymes**					
ALP	0.31 ± 0.03	0.39 ± 0.4 Δ	0.47 ± 0.03** Δ	0.32 ± 0.8** Δ	0.23 ± 0.04** Δ
ALT	0.25 ± 0.2	0.42 ± 0.2 Δ	0.48 ± 0.15** Δ	0.52 ± 0.15** Δ	0.56 ± 0.07** Δ
AST	2.54 ± 0.04	7.3 ± 0.07** ^Δ^	8.42 ± 0.11** ^Δ^	9.4 ± 0.04** ^Δ^	12.4 ± 0.03** ^Δ^
LDH	3.8 ± 0.51	3.1 ± 0.58 ^Δ^	4.7 ± 1.12** ^Δ^	8.3 ± 0.92** ^Δ^	5.8 ± 0.62** ^Δ^

Unit of measurements: WBCs (10^3/^mm^3^), RBCs (10^6/ml^), Hb (g/dL), HCT (%), MCV (%), MCH (%) MCHC (%), Platelets (10^3/mm3^). Values are expressed as Mean ± SD (n = 5 fish per treatment). Mean with ** expresses significant difference (p < 0.05) while Δ shows significant intergroup difference.

### 3.4 DNA damage

**Fig. 1:**
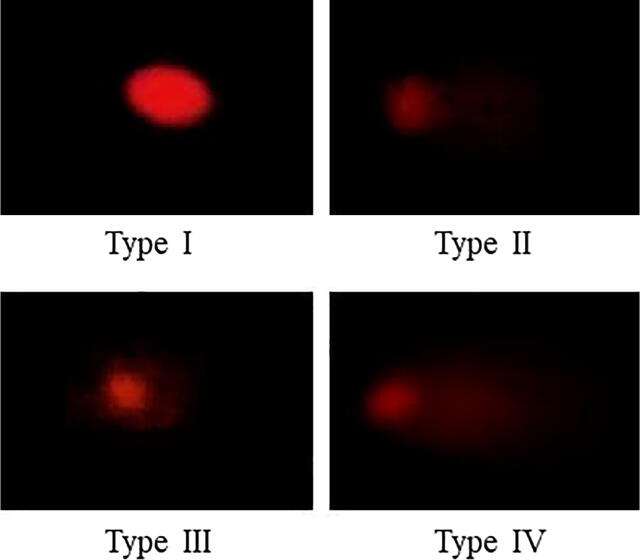
Representing DNA damage from type I-IV. Type I, low damage; type II, moderate damage; type III, high damage; type IV, complete damage.

After the exposure of *S. plagiostomus* to different concentrations of atrazine, significantly (p < 0.05) higher DNA damage was observed in erythrocytes counts in all experimental groups compared to the control group. Detail of DNA damage that occurred in treated groups is presented in Fig. 1 and Table 3.

**Table 3:** DNA damaged in *Schizothorax plagiostomus* exposed to atrazine.

**Concentration**	**DNA Damage (%)**			
	**Type I**	**Type II**	**Type III**	**Type IV**
1 ppm	30.00 ± 2.00** ^Δ^	21.33 ± 1.15**	16.67 ± 2.31** ^Δ^	9.33 ± 1.15**
2 ppm	20.00 ± 2.00**	22.00 ± 2.00** ^Δ^	15.33 ± 1.15** ^Δ^	18.00 ± 4.00** ^Δ^
3 ppm	28.00 ± 1.11** ^Δ^	7.33 ± 1.15** ^Δ^	7.33 ± 1.15**	6.67 ± 1.15** ^Δ^
4 ppm	32.00 ± 4.00** ^Δ^	12.00 ± 2.00**	10.00 ± 2.00** ^Δ^	5.33 ± 1.15** ^Δ^

Mean with ** expresses significant difference (p < 0.05) while Δ shows significant intergroup difference.

## 4 Discussion

This research is conducted to evaluate the adverse effects of atrazine on the hematology, biochemistry and genotoxicity of *S. plagiostomus*.

Tambaqui (*Colossoma macropomum*) showed irregular behavioral changes such as tiredness, balance losing, opercula activities frequency swelling and lips wideness proliferation when exposed to atrazine for 48 h for the dose of 20–25 mg L^−1^ (Chapadense et al., 2009). Comparable tendencies associated with inept performance are also reported in *Channa punctatus* exposed to the subtoxic amount of atrazine to the dose of 4.2–10.6 mg L^−1^ for 96 h (Nwani et al., 2010). In the present study irregular behavior including reduced responses, irregular swimming and loss of equilibrium were observed in all experimental groups. Mortality was observed only in the experimental group IV having the concentration of atrazine (4 ppm).

When fish is exposed to a stressor like pesticide exposure, it tries to adapt itself to that stressful environment. Blood is among the significant pathophysiological indicators of the body. Consequently, hematology and biochemistry alterations might be used as key indicators of detecting the health status of fish species facing pesticide stress (Adhikari et al., 2004, Evans and Claiborne, 2005). Atrazine is toxic; often bioaccumulative and persistent (Fernando et al., 1992). *C. carpio* exposed to atrazine result in alteration of blood parameters i.e, red blood cells amount (−63.17%), HBG (−27.35%), blood glucose (6.78%) and total protein (−18.73%) amounts become decline comparing with control group while white blood cells amount (+3.73%) improved. Changes in hematological and biochemistry were found significant (p < 0.05). White blood cell amount was not apparently altered (Ramesh et al., 2009). Hussein et al. (1996) stated that red blood cells amount, HBG amount and HCT level declined in *Oreochromis nitoticus* and *Chrysichthyes auratus* after exposure to 3 and 6 mg/l atrazine. Puigdoller et al. (2007) described the substantial rise in HCT level of Atlantic salmon after exposure to atrazine. Prasad et al. (1991) reported the damage of gill lamellae which sources reduced respiratory volume in Tilapia mosambica after exposure to 1.1 mgl^−1^ atrazine. Horn and Hanke (1980) reported a decrease in the number of erythrocytes of *C. carpio* when exposed to 0.1 mg/l ATR. Ventura et al. (2008) resulted in a great occurrence of micronuclei and nuclear aberrations in *O. niloticus* after exposure to diverse doses of atrazine. A declined level of erythrocyte was reported in fishes exposed to traumatic situations. Variations in erythrocyte amount advocate a reparation of oxygen shortage in the body because of gill destruction and the level of variations demonstrate a discharge of erythrocytes from the blood (Drastichova et al., 2004). The reticence of erythropoiesis and rise in the level of erythrocyte demolition in hematopoietic structures is the reason for the decline in red blood cell amount (Joshi et al., 2002). Rehwoldt (1978) presented a noteworthy decline in red blood cells, Hb and PCV in fishes exposed to atrazine and designated the noxious outcome of atrazine on liver, kidney and spleen. In our study exposure of *Schizothorax plagiostomus* to atrazine caused significant changes in all hematological parameters. Those exposed to higher concentrations were highly affected. The noteworthy decline in HB, HCT, WBC, RBC, MCH, MCHC, monocytes and lymphocytes was detected in treated fishes whereas the nonsignificant decrease in HCT and platelets count was detected in the group exposed to 4 ppm.

The level of fish blood protein serum is an index of the general health condition of the fish. Hussein et al. (1996) found a significant reduction of blood serum glucose. This reduction might be the result of the toxicity of the toxin on the kidney of the fish (Braunbeck et al., 1989). In recent studies, the reduction of the glucose plasma level during toxicity might belong to hypoxic conditions resulting from the herbicide atrazine. Chatterjee et al. (2004) have indicated an increase in energy petition may upturn protein ingestion, where the protein is transformed into energy, and consequently protein serum may become reduced. Imbalance in the blood is an important index of kidney damage. Damage of the kidney causes increased kidney protein secretion into the bloodstream and may also lead to the reduction of protein serum in finger-sized fish. Hussein et al. (1996) have reported that reduction in the total protein of *Chrysichthyes auratus* and *Oreochromis niloticus* was because of globulin reduction which shows that the effect of the toxin atrazine is on the fish immunity system. Ramesh et al. (2009) reported fatal effects of atrazine on hematology of *Cyprinus carpio* and assumed that tested hematology factor intensities were altered considerably due to atrazine. Gluth and Hanke (1985) indicated that carp species placed in atrazine for 72 h in the proximity of 100 µg/L concentration, showed a substantial reduction in blood protein concentrations due to the thinning effect in the blood of fish group. In our study, significant changes were observed in calcium, phosphorus, lactate, albumin, total protein and glucose as well as in levels of LDH, ALP, AST and ALT. A significant decrease is observed in all electrolytes in the four experimental groups. A significant decrease is observed in all metabolites while cholesterol, creatinine and glucose activity were observed to be increased in all experimental groups. A significant increase was observed in ALT and AST. An increase in activity of ALP was observed in experimental groups (1 ppm and 2 ppm) and after that decrease is observed in experimental groups (3 ppm and 4 ppm).

Liver cells must be traced to understand the degree of mutilation in genomic contents, as the liver is a key organ accountable for xenobiotic digestion and consequently is very vulnerable to impairment because of the occurrence of such noxious elements (Rajaguru et al., 2003). The plasma cells confirmed genotoxicity of *P. lineatus* exposed to atrazine, as fish treated with high doses of atrazine for two investigational phases revealed elevated levels of DNA destruction in plasma cells. (Ventura et al., 2008) reported many doses of equal herbicide in *Oreochromis niloticus* and got genotoxic alteration plasma cells due to atrazine exposure of 72 h. DNA mutilation perceived in diverse cells of *P. lineatus* treated with atrazine was possibly not due to the oxidative source, as no upsurge in ROS startup was identified in the liver and perhaps might not have been identified in other organs. Oliveira-Brett and Silva (2002) reported that triazine herbicides, like atrazine, have the ability to have the direct bond between adenine and guanine in DNA with the mechanism of intercalation and adduct construction. The principle technique to prevent damage of DNA is linking a xenobiotic to glutathione, created by the mechanism of glutathione S- transferase (Chakraborty et al., 2009). In the present study, it was found that atrazine has caused DNA damage in *S. plagiostomus*. Results obtained in this study show that atrazine even in low concentration (2 ppm, 3 ppm) was toxic to *S. plagiostomus* and has caused DNA damage. Exposure to 4 ppm caused complete DNA damage of *S. plagiostomus*. These results show that atrazine can promote DNA damage in *S. plagiostomus*.

## 5 Conclusion

From this study, it is evident that atrazine is lethal to *S. plagiostomus* and can cause alterations in biochemical and hematological parameters. Atrazine can also affect the behavior of *S. plagiostomus*. It is also found that exposure of *S. plagiostomus* to atrazine can promote DNA changes. It is concluded from this study that atrazine is toxic to aquatic fauna because causes alterations in hematological and biochemical parameters as well as DNA damage in *S. plagiostomus* at low concentration.

## Declaration of Competing Interest

The authors declares no conflict of interest.
